# Mutual information measure of visual perception based on noisy spiking neural networks

**DOI:** 10.3389/fnins.2023.1155362

**Published:** 2023-08-16

**Authors:** Ziheng Xu, Yajie Zhai, Yanmei Kang

**Affiliations:** School of Mathematics and Statistics, Xi’an Jiaotong University, Xi’an, China

**Keywords:** low-illumination image, spiking neural network, aperiodic stochastic resonance, mutual information, quantile threshold

## Abstract

Note that images of low-illumination are weak aperiodic signals, while mutual information can be used as an effective measure for the shared information between the input stimulus and the output response of nonlinear systems, thus it is possible to develop novel visual perception algorithm based on the principle of aperiodic stochastic resonance within the frame of information theory. To confirm this, we reveal this phenomenon using the integrate-and-fire neural networks of neurons with noisy binary random signal as input first. And then, we propose an improved visual perception algorithm with the image mutual information as assessment index. The numerical experiences show that the target image can be picked up with more easiness by the maximal mutual information than by the minimum of natural image quality evaluation (NIQE), which is one of the most frequently used indexes. Moreover, the advantage of choosing quantile as spike threshold has also been confirmed. The improvement of this research should provide large convenience for potential applications including video tracking in environments of low illumination.

## 1. Introduction

Stochastic resonance, initially proposed by European physicists in explaining the climatic switches (Benzi et al., 1981), is an essentially cooperative effect through which an external weak signal can be maximally amplified at a suitable amount of noise. This phenomenon is hard to be reproduced in climatic research but can be confirmed by various artificially designed experiments including crayfish (Douglass et al., 1993), shark (Braun et al., 1994), rat (Collins et al., 1996), cricket (Levin and Miller, 1996), optical material (Dylov and Fleischer, 2010) and human (Winterer et al., 1999; Zeng et al., 2000). The experiments successfully revealed that noise can play a potential but positive role in neural information processing, and further encouraged extensive theoretical progress, such as noise enhanced weak signal detection (Kang et al., 2005; Sun et al., 2019; Kang et al., 2022), noise facilitated information coding (Du et al., 2010; Nakamura and Tateno, 2019; Guan et al., 2021) and noise enhanced chaos control (Lei et al., 2017). Nowadays how to utilize noise for developing novel brain-like algorithms, such as visual perception (Simonotto et al., 1997; Fu et al., 2020; Xu et al., 2022) and epileptic diagnosis preprocessing (Shi et al., 2023), has attracted more and more interest in the current age of artificial intelligence.

Exploring the neural mechanism and algorithm design of visual perception is a long-standing topic in the field of neuroscience (Shapley and Hawken, 2002; Chen and Gong, 2019; Dijkstra et al., 2019). The visual perception in a general sense refers to the process of organizing, identifying, and interpreting visual information in environmental awareness and understanding (Yang et al., 2021), while in a narrow sense it means the enhancement of image contrast (Rafael and Woods, 2002). There are scenarios where pictures of high contrast are hard to be captured in a dark or low-illumination environment, such as in the cosmic exploration, in the battle front and in the deep-sea exploration. The traditional techniques (Land, 1997) for image enhancement are based on Retinex theory, where actual color sensations are assumed related to the intrinsic reflectance of objects. It is generally regarded that there are mainly two disadvantages of Single-Scale Retinex algorithm: one is that halo would be prone to occur in the transition field between strong light and shadow, and the other is that the image would be relatively dark after enhancement, so the Multi-scale Retinex algorithms (Rahman et al., 2004; Wang et al., 2021) have been proposed. Here, let us skip to assess the advantage or disadvantage of the improved variants, but choose to develop a different method based on different physical principles, namely stochastic resonance and spiking neuron models. In fact, the method proposed in this paper is an important development of our previous stochastic resonance-based spiking neural network methods in measure index, which is critical for identifying a target image.

How to effectively evaluate the perceptual quality of visual contents, such as image, is actually a long-standing issue. There are generally two categories of assessment indexes: the full-reference evaluation metric *via* the no-reference evaluation metric. The former category claims that the quality of the distorted image could be measured through comparing with a naturalistic reference image, including peak signal to noise ratio (Hore and Ziou, 2010) and structural similarity (Wang et al., 2004). The latter category covers perceptual quality metric (PQM) (Wang et al., 2002) and natural image quality evaluation (NIQE) (Mittal et al., 2013). As is known, PQM mainly concerns the blurring and blocking effect of a JPEG format image, while NIQE evaluates an image by calculating its distance from a fitted high-quality image. Nevertheless, when the both indices are applied to stochastic-resonance based visual perception algorithm design, it was found that the dependence of PQM on noise intensity tends to be too flat to pick out the best enhanced image (Fu et al., 2020), while the evolution of NIQE *via* noise intensity tends to have strong fluctuations due to the unpredictability in Gaussian distribution fitting and perturbation of external noise. It is these insufficiencies that motivate us to try a different measure from the viewpoint of information theory.

Our inspiration comes from the mutual information measure of aperiodic stochastic resonance (Collins et al., 1996; Levin and Miller, 1996; Patel and Kosko, 2008; Kang et al., 2021). Note that the external coherent input to stochastic resonant systems can be periodic or aperiodic, when it is aperiodic, the resonant phenomenon is specifically named as aperiodic stochastic resonance. In case of the aperiodic stochastic resonance, the quantifying indexes based on the mechanism of frequency matching, such as the signal-to-noise ratio and the spectral amplification factor, are no longer appropriate, and instead the input–output mutual information is a suitable choice for describing this matching mechanism of shape similarity. Following the investigation of aperiodic stochastic resonance (Kang et al., 2021), no matter how weak an external aperiodic signal is, it can always be maximally amplified by suitable amount of noise. Note that an image stimulus is such a typical aperiodic signal, thus the images of low contrast or illumination should always be optimally enhanced by an optimal dose of noise. This means there exists an optimal noise level at which the mutual information between the dark image and the target image can attain its maximum. As for whether the measure of mutual information is really a better choice for visual perception design, the comparison with NIQE should tell everything.

The paper is organized as follows. In Section 2 the phenomenon of aperiodic stochastic resonance in spiking neural networks consisting of integrate-and-fire neurons are exhibited within the information frame as preliminary. In Section 3 an improved visual perception algorithm is proposed based on the principle of aperiodic stochastic resonance. When applying the algorithm to both grayscale image and color image of low contrast, the reliability of the algorithm is verified. Different threshold strategies are also compared and the robustness of the mutual information measure is disclosed. Conclusions are finally drawn in Section 4.

## 2. Aperiodic stochastic resonance in an integrate-and-fire neural network

**Figure 1:**
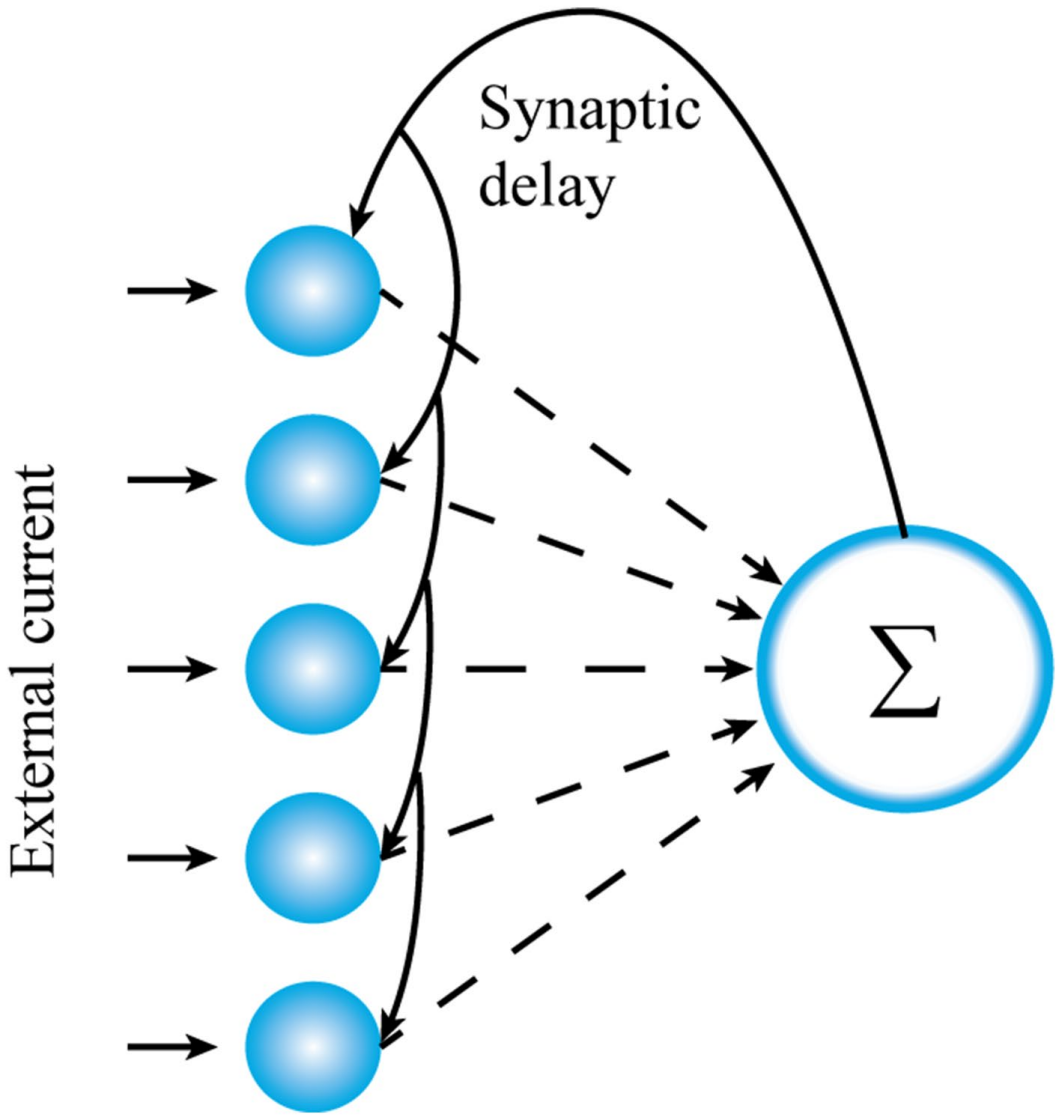
The network topology of the present study with each blue circle standing for one node neuron.


To enhance the plausibility of the subsequent visual perception algorithm, let us demonstrate here the principle of aperiodic stochastic resonance. Without loss of generality, let us continue to consider the spiking network consisting of the conductance-based fully connected integrate-and-fire neurons (Xu et al., 2022), as shown in Figure 1, with each neuron standing for one photoreceptor cell in the low illumination environment. The network is governed by the following Langevin equations

(1a) $$\begin{array}{l} C_{m}\frac{dV_{i}\left(t\right)}{dt}=-g_{l}\left(V_{i}\left(t\right)-V_{L}\right)-I_{syn,i}\left(t\right)\\ \;\;\;\;\;\;\;\;\;\;\;\;\;\;\;\;\;\;\;\;\;\;\;\;\;\;+I_{ext,i}\left(t\right),\;\;V_{i}\left(t\right)<V_{th},\;\;1\leq i\leq N \end{array}$$

(1b) $$I_{syn,i}\left(t\right)=g_{s}\left(V_{i}\left(t\right)-E_{syn}\right)\overset{N}{\underset{j=1}{\sum }}w_{ij}s_{j}\left(t\right)$$

(1c) $$s_{j}\left(t\right)=\underset{t_{j,k}}{\sum }e^{\left(-\left(t-t_{j,k}-\tau_{s}\right)/\tau \right)}\delta \left(t-t_{j,k}\right)$$

where $V_{i}\left(t\right)$ is the membrane potential of the *i*th neuron at time *t*, $C_{m}$ is the membrane capacitance, $g_{l}$ is a leaky conductance, $V_{L}$ is a leaky voltage, $I_{syn,i}\left(t\right)$ is the synaptic current at time *t* from other neurons in the network, and $I_{ext,i}\left(t\right)=S\left(t\right)+\sqrt{2D}\xi_{i}\left(t\right),1\leq i\leq N$ denotes an external injected current. In the external current, S(t)∈{A,B} is a binary signal representing an external visual stimuli, with P(S(t)=A)=p and P(S(t)=B)=1−p with p∈(0,1) , $\xi_{i}\left(t\right)$ is Gaussian white noise of noise intensity D and describing the external fluctuations satisfying $\langle \xi_{i}\left(t+s\right)\xi_{j}\left(t\right)\rangle =\delta_{ij}\delta \left(s\right)$ for 1≤i,j≤N . Here, δ(⋅) is Dirac function δ(⋅) while $\delta_{ij}$ is Dirac notation such that $\delta_{ij}=1$ if i=j and $\delta_{ij}=0$ otherwise. In Eq. (1b), $g_{s}$ is the synaptic conductance, $E_{syn}$ the synaptic reversal potential, $w_{ij}$ the synaptic weight between neuron *i* and neuron *j*, τ is the synaptic constant, $\tau_{s}$ the synaptic delay, $t_{j,k}$ the *k*th spiking time of the neuron *j*, and $s_{j}\left(t\right)$ is the fractions of open synaptic channels of the *j*th neuron at time *t*. Once the membrane potential $V_{i}\left(t\right)$ reaches the threshold potential $V_{th}$ from below, a spike is emitted and the membrane potential is immediately reset to the resting potential $V_{r}$ and restarts a time-dependent evolution following Eq. (1a) after a short refractory period $\tau_{ref}$ . For the sake of simplicity, we set $\tau_{ref}=0$ by ignoring the influence of refractory period.Note that the train of spikes is the main carrier for neural information, thus the output response of the *i*th neuron and the neural network can be, respectively, denoted as


(2) $$y_{i}\left(t\right)=\underset{k}{\sum }\delta \left(t-t_{i,k}\right),\;\;y\left(t\right)=\frac{1}{N}\underset{i,k}{\sum }\delta \left(t-t_{i,k}\right)$$

We remark that the spike train $y_{i}\left(t\right)$ can be acquired by Euler-Maruyama scheme, namely


(3) $$\begin{array}{l} C_{m}\Delta V_{i}^{n+1}=-\left(g_{l}\left(V_{i}^{n}-V_{L}\right)-I_{syn,i}\left(t_{n}\right)-I_{sig}\left(t_{n}\right)\right)\Delta t_{n}\\ \;\;\;\;\;\;\;\;\;\;\;\;\;\;\;\;\;\;\;\;\;\;\;\;+\sqrt{2D\Delta t_{n}}r_{i}^{n},\;\;1\leq i\leq N \end{array}$$

Here the superscript *n* denotes the *n*th iteration. $V_{i}^{n}=V_{i}\left(t_{n}\right)$ , $I_{sig}\left(t_{n}\right)=S\left(t_{n}\right)$ , $\Delta t_{n}=t_{n+1}-t_{n}$ is time step, $\Delta V_{i}^{n}=V_{i}\left(t_{n+1}\right)-V_{i}\left(t_{n}\right)$ and $r_{i}^{n}∼N\left(0,1\right)$ is a normal distributed pseudo random number. Note that Gaussian white noise is the formal derivative of Wiener process. Since the Wiener process is of independent increments, these mutually independent pseudo random numbers at each iteration are also statistically independent for different iterations.

Note that noise can play a beneficial role in improving neural information encoding through the mechanism of stochastic resonance (Rizzo, 1997). Particularly, the input–output mutual information is suitable for acting as a quantifying metric for aperiodic stochastic resonance (Kang et al., 2021). For the sake of completeness, let us explain how to calculate the input–output mutual information for the system (1). Let I(X,Y) be the mutual information of discrete random variables X and Y with values in finite sets χ and γ , then


(4) $$I\left(X,Y\right)=H\left(Y\right)-H\left(Y|X\right)=\underset{x\in \chi }{\sum }\underset{y\in \gamma }{\sum }p\left(x,y\right)\log\frac{p\left(x,y\right)}{p\left(x\right)p\left(y\right)}$$

where $H\left(Y\right)=-\underset{y\in \gamma }{\sum }p\left(y\right)\log\left(p\left(y\right)\right)$ and $H\left(Y|X\right)=-\underset{x\in \chi ,y\in \gamma }{\sum }p\left(x,y\right)\log\left(p_{Y|X}\left(y|x\right)\right)$ are the entropy and the conditional entropy (Cover and Thomas, 2006; Yarrow et al., 2012), respectively. We follow the existing procedure (Kang et al., 2021) to calculate the mutual information I(S(t),y(t)) between the binary input S(t) and the population firing output y(t) . As seen from Eq. (4), I(S(t),y(t)) is a mathematical expectation of the form


(5) $$I\left(S\left(t\right),y\left(t\right)\right)=E\left(\log\frac{p\left(s,y\right)}{p\left(s\right)p\left(y\right)}\right)$$

Thus, we can repeat 1,000 trials to get an arithmetic average for an improved accuracy based on the law of large number.

Note that the entropy H(y(t)) quantifies the average uncertainty of a random variable y(t) , while the conditional entropy H(y(t)|S(t)) measures the average uncertainty associated with y(t) under the condition that the outcome of S(t) are known, thus I(S(t),y(t)) , as a measure of the shared information between the binary input and the population firing output, can be adopted as metric for the phenomenon of aperiodic stochastic resonance. When the input–output mutual information of the model (1) has a nonmonotonic dependence on noise intensity, it is usually said that the phenomenon of aperiodic stochastic resonance occurs (Collins et al., 1996). Particularly, when the input–output mutual information is maximized, the output signal should have a maximal resemblance in shape with the input signal.

**Figure 2:**
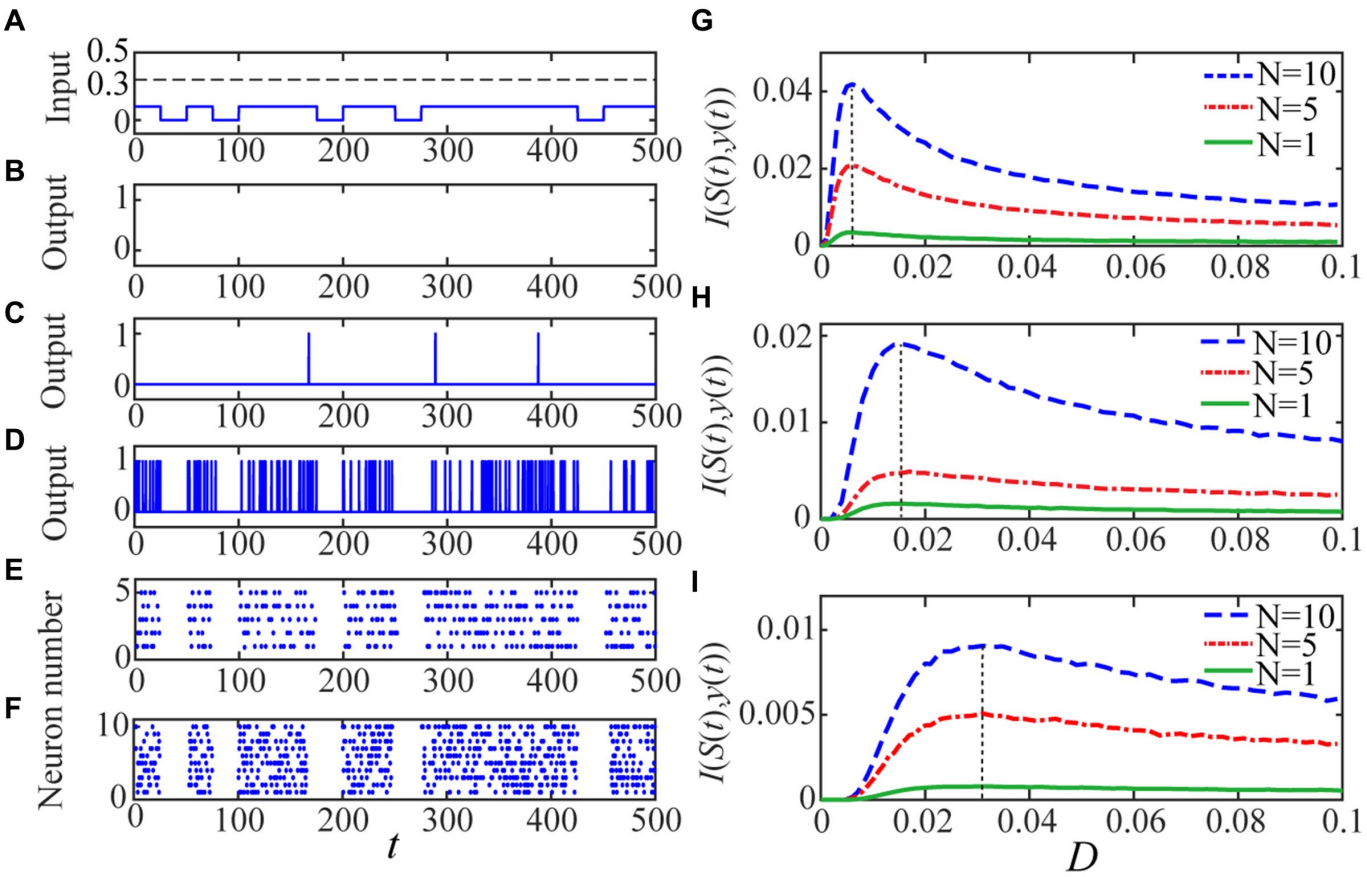
The quantized output and the input–output mutual information are displayed. The random binary signal is displayed in panel **(A)** with *A* =0.1, *B* =0 and *p* = 0.7. For given *V_th_* = 0.2, as the panel **(B)** shows there is no 1 in the quantized output for the single neuron when the noise intensity is vanishing since the input signal is subthreshold. As the noise intensity is increased, the quantized output becomes more visible and clearly, there is more input–output similarly for a single neuron with panel **(D)** *D* = 0.006 than the other level **(C)** *D* = 0.001. The resemblance becomes evident when **(E)** *N* = 5 and **(F)** *N* = 10 for the optimal noise intensity, which is the same for different network size under the identical spike threshold. The mono-peak curves of mutual information *I* (*S*(*t*),*y*(*t*)) via noise intensity with thresholds: **(G)** *V_th_* = 0.2, **(H)** *V_th_* = 0.3 and **(I)** *V_th_* = 0.4 signify the occurrence of aperiodic stochastic resonance for different size’ network. The other parameters are fixed as *V_re_* = 0, *gl* = *gs* = 1, *C*_m_ = 1, *E_syn_* = 0, τ_s_ = 1, τ_d_ = 0.5 and *T* = 500.

We consider a realistic inhibitory synaptic weight *w*_*ij*_ = −0.2 (Rolls et al., 2008) for 1≤i,j≤N to observe aperiodic stochastic resonance for both a single neuron and the spiking network. Since the binary input is subthreshold (Figure 2A), there is no spike emission from the single neuron in the absence of noise (Figure 2B). When small amount of noise is injected, the single neuron is activated with the help of noise but the resultant output response is obviously different from the binary input signal in shape (Figure 2C). When the noise intensity is increased to a proper level where the input–output mutual information attains a peak value (Figure 2G), the resemblance between the output response and the input binary signal is greatly improved, as shown in Figure 2D. Note that the perception function of the brain is generally implemented at population level, while the effect of stochastic resonance can be enhanced by uncoupled array or coupled ensemble (Nakamura and Tateno, 2019; Sun et al., 2019). Thus, in order to simulate this synergetic effect of system size on the aperiodic stochastic resonance, we also show the shape similarity by raster plots for N=5 (Figure 2E) and N=10 (Figure 2F). From these pictures it is clear that the shape similarity significantly increases as the network size grows, thus a larger network should be necessary for the subsequent visual perception design. We emphasize that all the shape similarities are selected when the mutual information of Figures 2D–F reaches maximum. This demonstrates that the input–output mutual information as function of noise cannot unlimitedly increase, and therefore there exists an optimal noise intensity at which the weak input signal can be best detected. Additionally, from Figures 2G–I, it is clear that the aperiodic stochastic resonance has a strong dependence on the spike threshold: the lower the threshold, the prominent the resonant effect. This point has an important inspiration: a suitable threshold should be chosen so that an optimally enhanced image can be acquired. In fact, the spike threshold in real neurons has certain circadian rhythm and self-adaptability, so it can be lower in a dimmer environment (Destexhe, 1998; Taillefumier and Magnasco, 2013).

As the last paragraph, let us emphasize that the shape similarity measured by the mutual information can be further improved by increasing the network size, as shown by Figures 2D–F, but the improvement should have limitation following the law of large number. In fact, According to the previous investigations, whether the quantifying index is the spectral amplification factor (Fu et al., 2020) or the signal to noise ratio (Xu et al., 2022), it cannot be improved infinitely; on the contrary, the limit level can be achieved with a network size N≤50 for the aperiodic binary S(t) . Noting the image signal is more complex than the binary input, we choose N=300 in the subsequent algorithm implementation so that the benefit of network size can be maximally utilized. This should imply the following fact. Although there are over 100 million photoreception neurons for one normal person (Rieke and Baylor, 1998), not all of them participate in the perception task: the more complex the stimuli are, the more neurons are involved.

## 3. Visual perception algorithm and mutual information measure

Noise is not only ubiquitous in nervous systems but can play a positive role in neural information processing (Rizzo, 1997). As illustrated by the last section, noise can potentially assist human being in detecting weak aperiodic stimuli. Note that an image of low contrast is such a typical stimulus, thus some biologically plausible visual perception algorithms (Fu et al., 2020; Xu et al., 2022) have been proposed by combining the basic biophysical process behind visual perception, which includes three stages of encoding, decoding and integrating, with the principle of aperiodic stochastic resonance. Nevertheless, as mentioned in the introduction section, one of the most commonly used assessment indices, namely NIQE (Mittal et al., 2013; Xu et al., 2022), always gives rise to strong fluctuations as noise intensity increases. To overcome this insufficiency in the existing algorithms, we aim to present an improved algorithm within the frame of information theory.

### 3.1. Grayscale image enhancement

When light enters the eye, the photoreceptors in the retina transform the optical signal into an electrical signal through an inherent encoding process participated by rod cells. Note that there are two kinds of photoreceptors in the retina: rods and cones. The cones in charge of color are active to bright light while the rods in charge of profile are more sensitive to dim light. In the low-illumination environment, the rhodopsin in rod cells can decompose itself under the light stimulation so that the light signals can be transferred into electrical signals (Hartong et al., 2006). As a result, human can discern to certain extent the profile of hidden objects in dim surroundings. Thus, we use the spiking neural network (1) to simulate the rod cells and their feedback interaction (Xu et al., 2022), while the aperiodic binary input can be replaced by the weak image stimulus.

In the encoding state, let Gray be an *M*-dimensional grayscale matrix of a white-black image, with each pixel representing an illuminance value. Note that all the rods in the network are assumed to focus on the same image stimuli, thus the illuminance matrix can be received by every rod. Let $V_{i}^{m,n}\left(t\right)$ represents the time-dependent potential response of the *i*th rod cell when it receives the pixel Gray(m,n) with 1≤m,n≤M, then the spiking neural network (1) can be rewritten into


(6a) $$\begin{array}{l} C_{m}\frac{d}{dt}V_{i}^{m,n}\left(t\right)=-g_{l}\left(V_{i}^{m,n}\left(t\right)-V_{L}\right)+I_{syn,i}^{m,n}\left(t\right)\\ \;\;\;\;\;\;\;\;\;\;\;\;\;\;\;\;\;\;\;\;\;\;\;\;\;\;\;\;\;\;\;\;\;\;\;+Gray\left(m,n\right)+\sqrt{2D}\xi_{i}^{m,n}\left(t\right) \end{array}$$

(6b) $$I_{syn,i}^{m,n}\left(t\right)=g_{s}\left(V_{i}^{m,n}\left(t\right)-E_{syn}\right)\overset{N}{\underset{j=1}{\sum }}w_{ij}s_{j}^{m,n}\left(t\right)$$

(6c) $$s_{j}^{m,n}\left(t\right)=\underset{t_{j,k}}{\sum }\exp\left(-\left(t-t_{j,k}^{m,n}-\tau_{l}\right)/\tau \right)\delta \left(t-t_{j,k}^{m,n}\right)$$

where $\xi_{i}^{m,n}\left(t\right)$ is Gaussian white noise satisfying $\xi_{i}^{m,n}\left(t+s\right)\xi_{j}^{m,n}\left(t\right)=\delta_{ij}\delta \left(s\right)$ for i,j=1,2,…,N . Once $V_{i}^{m,n}\left(t\right)$ reaches the spike threshold $V_{th}$ from below, an action potential is generated by neuron *i* emits and then the membrane potential is immediately reset to the resting potential $V_{r}$ , from where a new cycle of evolution restarts. In the encoding phase, $Index_{i}\left(m,n\right)\in \left\{0,1\right\}$ , the (*m*, *n*) element of the spike matrix $Index_{i}$ is adopted to mark whether the *i*th neuron has generated spikes during given time span or not. That is, $Index_{i}\left(m,n\right)=1$ if there is spike generation and $Index_{i}\left(m,n\right)=0$ otherwise. Here, the time span T is taken as one millisecond to approximate the time cost by a gaze from a normal person and specifically, we emphasize that the grayscale image is taken as a continuous input during the whole time span. That is, every rod cell receives the same constant grayscale matrix. We emphasize that the encoding scheme follows from the previous algorithms (Fu et al., 2020; Xu et al., 2022), which also have certain association with image reconstruction algorithms (Roy et al., 2019; Li et al., 2022). Noting that there are N neurons in the network, there should be N such 0–1 counting matrices altogether. With these spike matrices available, ganglion cells in the last segment of the retina can then transmit the involving information to visual cortex for the next stage (Masland, 1996). For the sake of simplicity, we fix the neural network parameters $C_{m}=1$ , $g_{l}=g_{s}=1$ , $\tau_{s}=1$ , $\tau_{d}=0.5$ , $E_{syn}=0$ , $w_{ij}=-0.2$ , $V_{re}=0$ , T=1 and N=300 but leave $V_{th}$ and D tunable.

Note both the stage of decoding and the stage of integrating are implemented at the visual cortex. Since the binary spike trains are the main carrier of neural information (Strong et al., 1996), it is reasonable to assume that each encoded spike matrix should be decoded into a binary image. Then, the spike matrix $Index_{i}$ encoded by the *i*th neuron can be decoded as a gray image $Pic_{i}$ of element


(7) $$Pic_{i}\left(m,n\right)=\{\begin{array}{l} 0,Index_{i}\left(m,n\right)=0\\ 255,Index_{i}\left(m,n\right)=1 \end{array}$$

With all the decoded binary images available, an integrated grayscale image Pic can be obtained by an ensemble average, namely


(8) $$Pic\left(m,n\right)=\frac{1}{N}\overset{N}{\underset{i=1}{\sum }}Pic_{i}\left(m,n\right)$$

Considering the visual perception process is carried on in noisy environment, every binary image $Pic_{i}$ is actually a matrix-valued random variable. Thus, the treatment in Eq. (8) is somehow similar to the effect of large number law, which cancels out the influence of occasional factors by taking arithmetic average. In fact, this integration treatment has biophysical implication as well. As is known, a single visual cortex cell usually does not receive all the signals from photoreceptors but only a specifically dominated area (Lecun and Bengio, 1995). Thus, integrating over all the decoded binary images helps to assure that all the information of the visual content is processed.

The purpose of visual perception is to pick out the best enhanced image under the help of suitable amount of noise, but the noise intensity fixed in the above procedure is generally not optimal. Nevertheless, it is possible to capture an optimal value following the principle of aperiodic stochastic resonance. Let us measure the quality of the enhanced image by the input–output image mutual information, namely the mutual information between Gray and Pic . To this end, the pixels of Gray and Pic are divided into 255 bins of unit size: [0,1], …, [254,255] ; the frequency number of the pixels is counted in each bin and then the histograms for Gray and Pic are obtained; the joint histogram of Gray and Pic can be acquired in the same way. With these histograms to approximate the corresponding marginal distribution laws and the corresponding joint distribution law, the input–output image mutual information could be calculated by


(9) $$I\left(Pi\mathrm{c,}Gray\right)=\underset{Pic,Gray}{\sum }P\left(Pi\mathrm{c,}Gray\right)\log\left(\frac{P\left(Pi\mathrm{c,}Gray\right)}{P\left(Pic\right)P\left(Gray\right)}\right)$$

Then, by examining the nonmonotonic dependence of the image mutual information *via* the noise intensity, one can attain the optimal noise intensity, at which the enhanced image is exactly the target image of our visual perception algorithm. Note that the external noise level of a neural network can be self-adjusted by synaptic weights (Feng et al., 2006), thus it is also biologically plausible to optimize the enhanced image by an optimal noise intensity.

**Figure 3:**
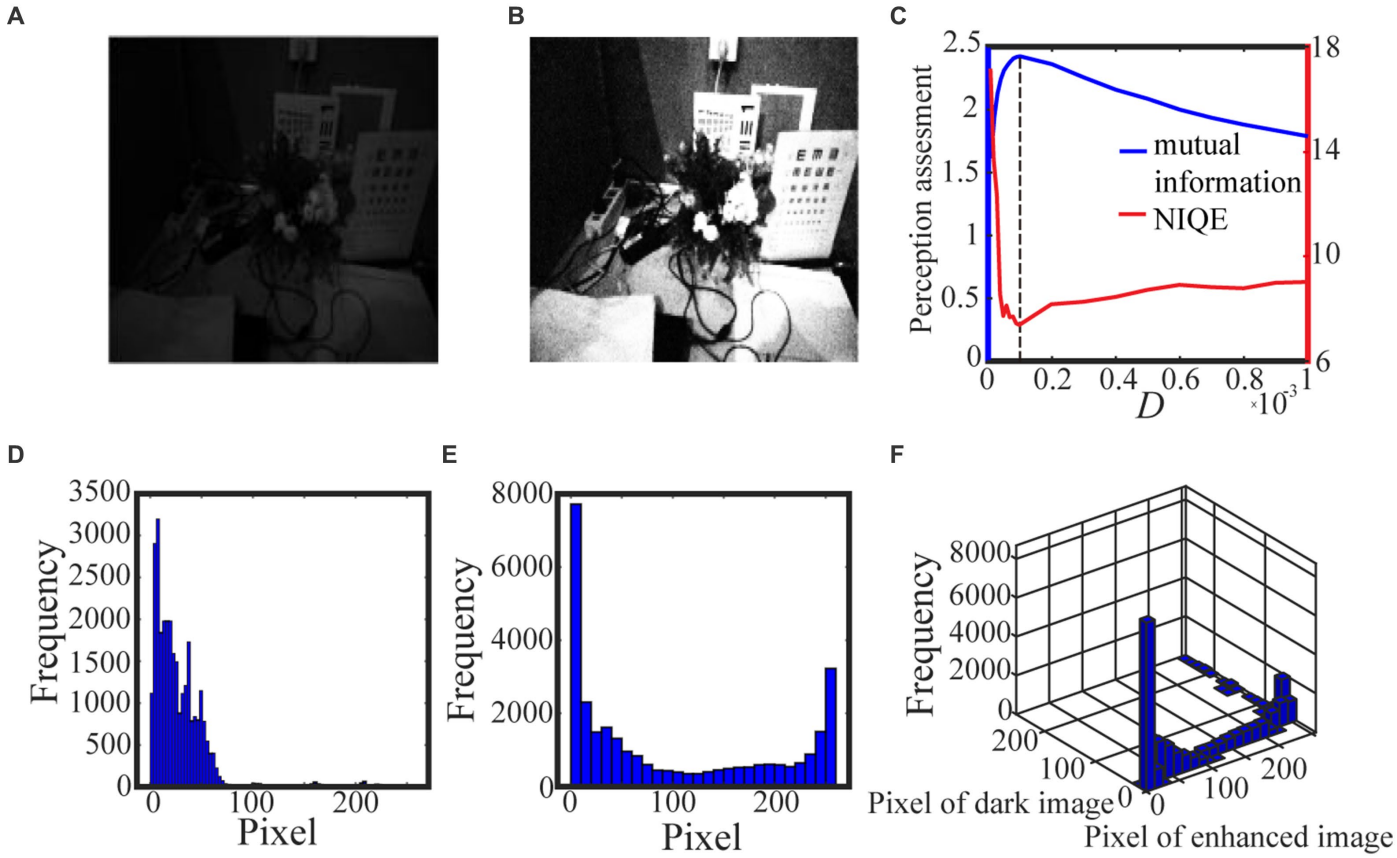
The low illuminance grayscale image in **(A)** is taken from the internet and the 0.5 quantile is adopted as the spike threshold. The marginal probability law of **(A)** is shown in **(D)** while the marginal probability law of the best enhanced image **(B)** selected by mutual information is shown in **(E)**. The joint probability law of **(A)** and **(B)** are shown in the two-dimensional histogram **(F)**. The quantifying indexes mutual information (blue solid line) and NIQE (red solid line) are plotted in **(C)** where the black dotted line denotes the optimal noise intensity.


**Figure 4:**
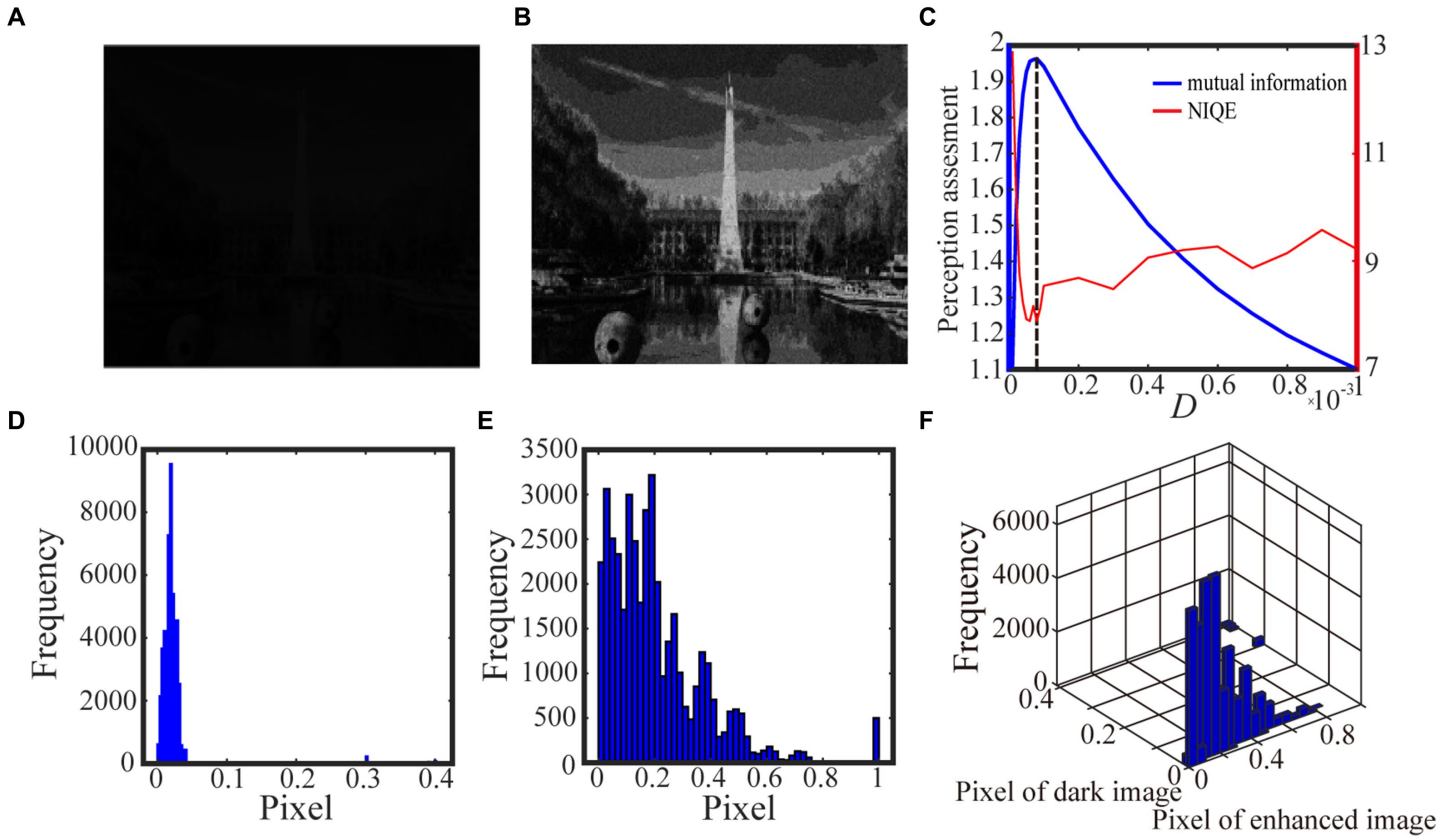
The darker grayscale picture in **(A)** is taken by us and the 0.6 quantile is taken as the spike threshold. The marginal probability law of **(A)** is shown in **(D)** while the marginal probability law of the best enhanced image **(B)** picked out by mutual information is shown in **(E)**. The joint probability law of **(A)** and **(B)** is shown in the two-dimensional histogram **(F)**. The perception assessments mutual information (blue solid line) and NIQE (red solid line) are plotted in **(C)** where the black dotted line denotes the optimal noise intensity.

Algorithm 1 summarizes the above procedure for visual perception, while Figures 3, 4 show the test results. For the test in Figure 3, we take 0.5 quantile as the spike threshold (Xu et al., 2022). The marginal histograms for the original picture (Figure 3A) and the target image (Figure 3B) are shown in Figures 3D,E, respectively. By comparing Figures 3D,E, it is clear that more pixels with relatively low grayscale value have been shifted to the area of relatively high grayscale value, which explains why the hidden objects such as flowers and visual charts can be revealed in the target image. Here, we emphasize that the nonmonotonic curve of the image mutual information *via* noise intensity in Figure 3C signifies the occurrence of aperiodic stochastic resonance) and the target image in Figure 3B is picked out from the peak value of the mutual information curve. In fact, our numerical experience has confirmed that a bit derivation from the optimal noise intensity can cause degradation in the quality of the enhanced image. The performance of algorithm 1 can be further confirmed by a darker original image. As shown in Figure 4, the 0.6 quantile is taken as the spike threshold. Analogously, by comparing the marginal histograms of the dark image (Figure 4A) and the target image (Figure 4B), as shown in Figures 4D,E, it is clear to see that the low-value pixels have been increased. Therefore, the tower, trees, the building and the reflection of the tower become discernable in the target image, which corresponds to the peak value of the mutual information curve. Note that the distribution of the natural image features is close to the generalized Gaussian distribution (Moorthy and Bovik, 2010), thus the parameters of the generalized Gaussian distribution fitted with the image to be enhanced should have certain distance of the parameters fitted with natural images. The distance is the so called NIQE, defined by Mittal et al. (2013)

(10) $$\mathrm{NIQE}=\sqrt{{\left(v_{1}-v_{2}\right)}^{T}{\left(\frac{\sum_{1}+\sum_{2}}{2}\right)}^{-1}\left(v_{1}-v_{2}\right)}$$

where $\nu_{1}$ and $\Sigma_{1}$ are the mean vector and the covariance matrix fitted with the natural images, respectively, and $\nu_{2}$ , $\Sigma_{2}$ are the mean vector and the covariance matrix fitted with the images to be enhanced, respectively. It is clear that the smaller NIQE, the better the image quality. Therefore the minimum of NIQE corresponds to the best enhanced image. As seen from noise intensity denoted by the dash line in Figures 3C and 4C, although the optimal noise intensities obtained from the two metrics are almost the same, the mutual information curves are smoother than the NIQE curves. This suggests that the image mutual information is a more appropriate metric for assessing the grayscale image quality.

**Table 1:** 

**Algorithm 1** Image enhancement for grayscale image**s**	
**Step1:**	Input the grayscale image Gray to the integrate and fire neuronal network under noise intensity D to get the matrix $Index_{i},i=1,\ldots ,N$ which stores the spiking information.
**Step2:**	Transfer $Index_{i},i=1,\ldots ,N$ to grayscale image $Pic_{i},i=1,\ldots ,N$
**Step3:**	Calculate $Pic=\frac{1}{N}\overset{N}{\underset{i=1}{\sum }}Pic_{i}$ , mutual information I(Pic,Gray) and NIQE
**Step4:**	Change *D* and repeat Step1 to Step 3 until the best enhanced image is selected

### 3.2. Color image enhancement

The color image of low-illumination (contrast) has much difference from the grayscale images, since several color channels are usually involved in given color space. As is known, there are distinct color spaces, such as Red-Green-Blue (RGB) space, Hue-Saturation-Value (HSV) space and Y-cbcr color space (Smith, 1978; Rafael and Woods, 2002; Benjamin et al., 2016; Wu et al., 2021), among which the RGB space is suitable for computer Graphics, the Y-cbcr space is good at discrimination of luminance and chrominance, while the HSV space is in line with the human visual perception system. Based on this consideration, we can transfer the signal from the RGB space into the HSV space. Since the illumination of a color image is overwhelmingly dominated by its value matrix, it is enough to enhance the value information for color image perception.

Let L be the value matrix. Following the general process of vision formation in low illuminance environment, we can adapt Algorithm 1 with the grayscale matrix Gray replaced by L so that the illumination information can be encoded by the rod cells on the retina. And then, the encoded spike matrices $Index_{i}$ for i=1,…,N can be transmitted to visual cortex and finally integrated into a binary image Img . As the value information L is the input image and Img is the output image, we can measure the input–output similarity by the image mutual information defined by


(11) $$I\left(Img,L\right)=\underset{Img,\;\;L}{\sum }P\left(Img,L\right)\log\left(\frac{P\left(Img,L\right)}{P\left(Img\right)P\left(L\right)}\right)$$

**Figure 5:**
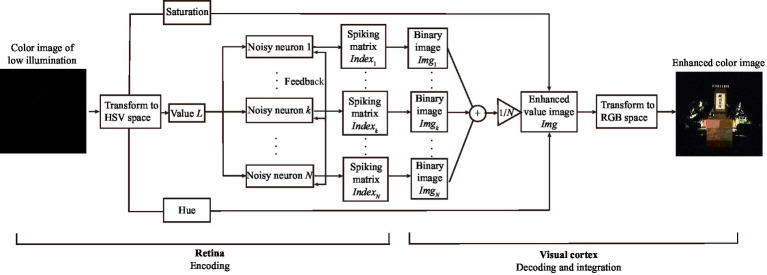
Schematic diagram of color image enhancement algorithm.

Here P(L) and P(Img) are the marginal distribution laws for the original value matrix and the enhanced image which is noise intensity dependent, respectively, and P(Img,L) denotes the joint distribution law. Again, these distribution laws can be approximated by their histograms, thus the input–output image mutual information is still easy to be obtained. Once the best enhanced value matrix is attained, an inverse transform from the HSV space into the RGB space can generate a best enhanced color image, which is the target image for color vision perception. The main procedure has been summarized in Algorithm 2. To enhance the intuitiveness, the flowchart of the algorithm is also displayed in Figure 5.

**Figure 6:**
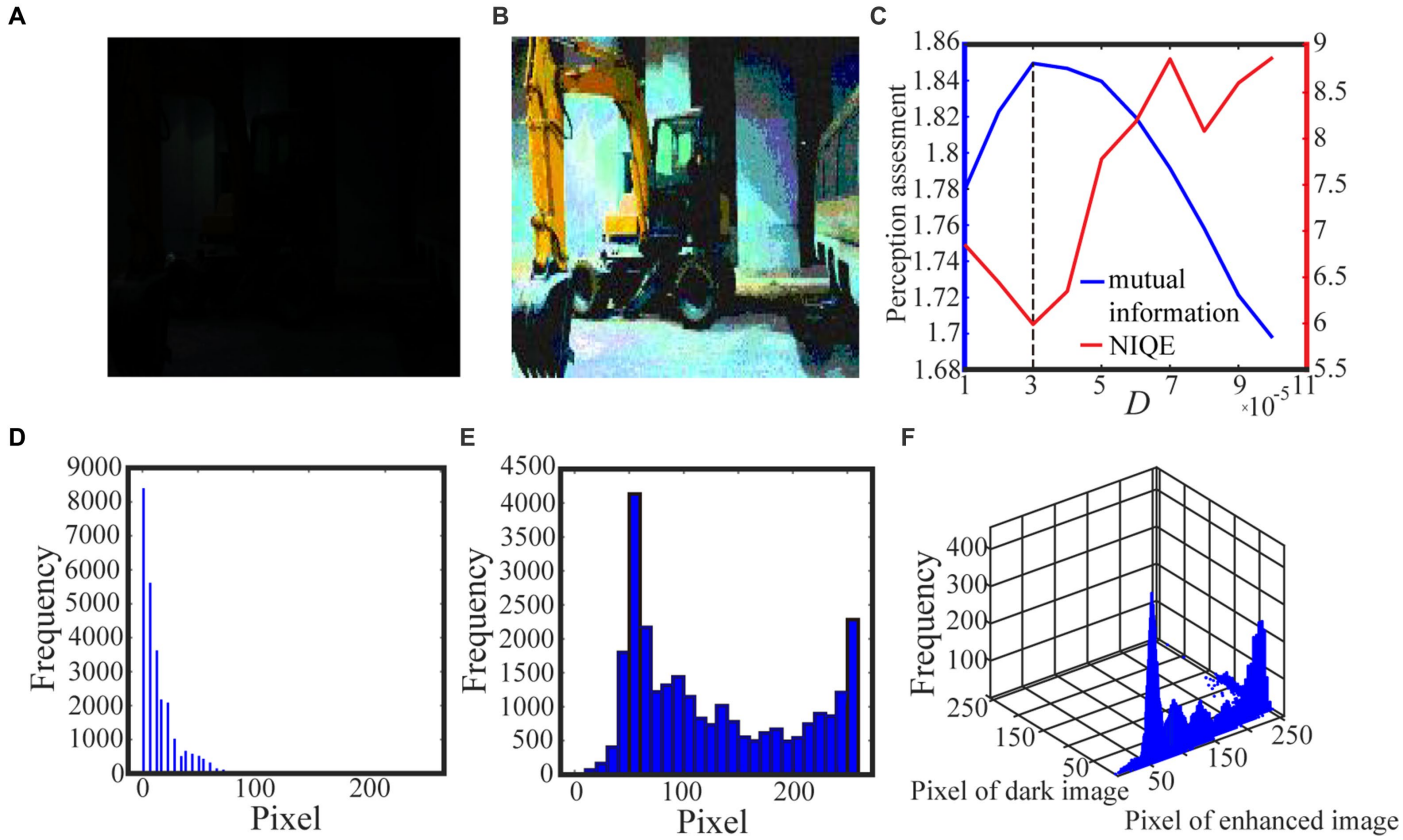
The dark color image in **(A)** is taken from the internet and the 0.6 quantile is used as the spike threshold. The marginal probability law of **(A)** is shown in **(D)** while the marginal probability law of best enhanced color image **(B)** selected by mutual information is shown in **(E)**. The joint probability law of **(A)** and **(B)** are shown in the two-dimensional histogram **(F)**. The quantifying indexes mutual information (blue solid line) and NIQE (red solid line) are plotted in **(C)** where the black dotted line denotes the optimal noise intensity corresponding to the maximal mutual information.

**Figure 7:**
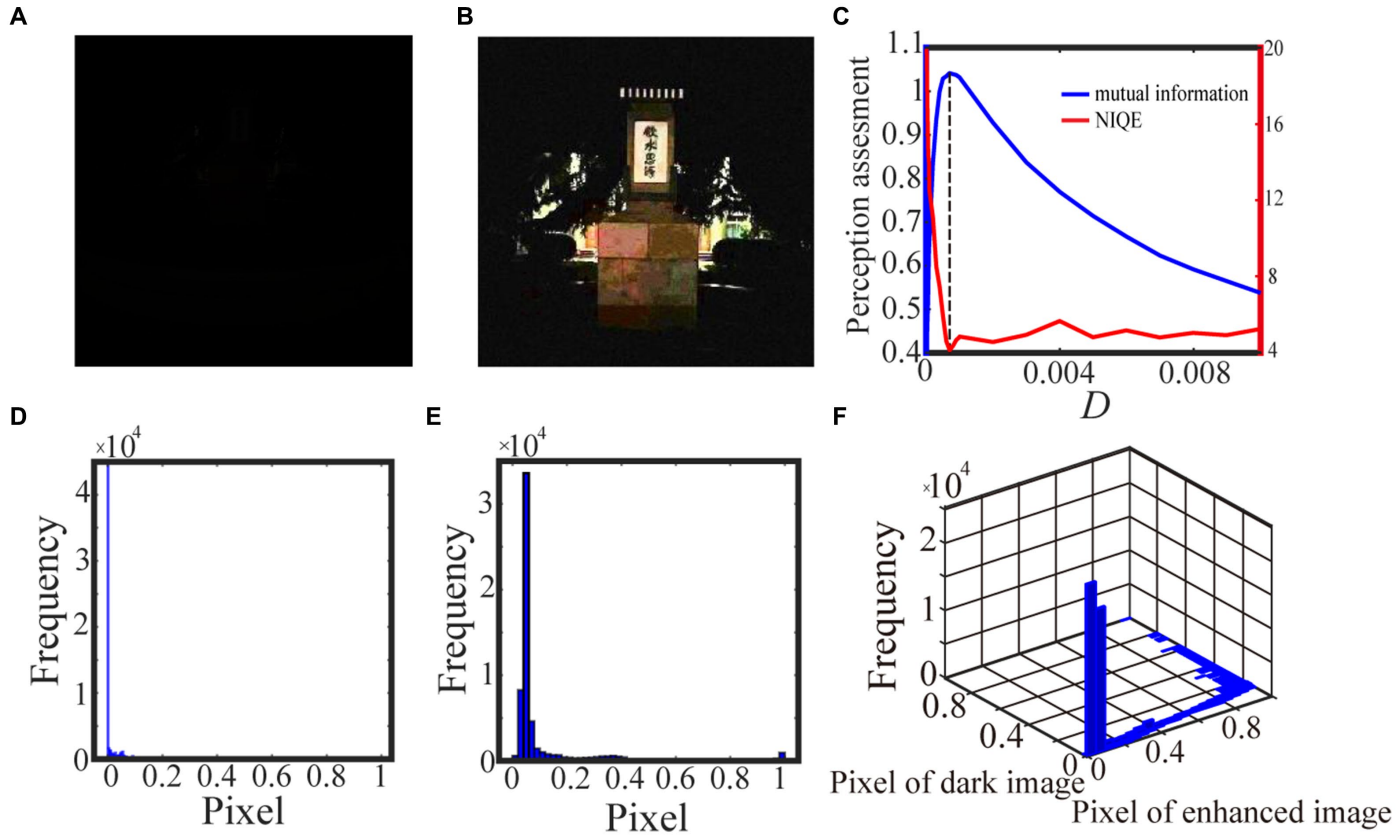
The low illuminance color image in **(A)** is taken by us and the 0.9 quintile is the spike threshold. The marginal probability law of **(A)** is shown in **(D)** while the marginal probability law of best enhanced color image **(B)** selected by mutual information is shown in **(E)**. The joint probability law of **(A)** and **(B)** are shown in the two-dimensional histogram **(F)**. The quantifying indexes mutual information (blue solid line) and NIQE (red solid line) are plotted in **(C)** where the black dotted line denotes the optimal noise intensity corresponding to the maximal mutual information.

We exhibit two test results with low-contrast color images acquired from the internet and the real world in Figures 6 and 7, respectively. For the test in Figure 6, the 0.6 quantile is taken as the spike threshold. As shown from Figure 6C the nonmonotonic curve of the image mutual information via noise intensity again verifies the occurrence of aperiodic stochastic resonance) and the target image in Figure 6B is picked out from the peak value of the mutual information curve. By comparing the marginal histograms Figures 6D,E for the original picture Figure 6A and the target image Figure 6B, it is clear that more pixels with relatively low illumination value have been shifted to the area of relatively high illumination value, which again explains why the hidden objects such as a bus and an excavator become exposed in the target image. It is clear from Figure 7 that some details of the target image selected by the peak value of the mutual information curve become visible, such as the characters on the monument. Here, we emphasize that the image is indeed enhanced, although most of the pixels of the marginal histogram in Figure 7E are still relatively low. The reason behind this observation is that most of the pixel values are zero since no color and illuminance information can be available from this totally dark environment. Here we also would like to emphasize that the principle of stochastic resonance is powerful in enhancing weak signal, no matter how weak it is, but the weak signal must exist at first. Therefore, we claim that Figure 7B is already the best enhanced image and the corresponding noise intensity is optimal, as revealed by the perception indexes shown in Figure 7C. Finally, from Figures 6,7C we see again that the optimal noise intensities obtained from the two metrics are coincident and the mutual information curves are smoother than the NIQE curves. This again illustrates that the image mutual information is a more appropriate metric for assessing the quality of an enhanced color image.

**Table 2:** 

**Algorithm 2** Image enhancement for color images	
**Step1:**	Transform the image from RGB space to HSV space.
**Step2:**	Input the value matrix *L* to the integrate and fire neural network under noise intensity D to get the matrix $Index_{i},i=1,\ldots ,N$ which stores the spiking information.
**Step3:**	Transfer $Index_{i},i=1,\ldots ,N$ to grayscale image $Img_{i},i=1,\ldots ,N$ .
**Step4:**	Calculate $Img=\frac{1}{N}\overset{N}{\underset{i=1}{\sum }}Img_{i}$ , mutual information I(Img,L) and NIQE .
**Step5:**	Change *D* and repeat Step2 to Step 4 until the best enhanced value matrix is selected.
**Step6:**	Combine the best enhanced value matrix with hue matrix and saturation matrix and transform it from HSV space into the RGB space.

### 3.3. Effect of threshold strategy

**Figure 8:**
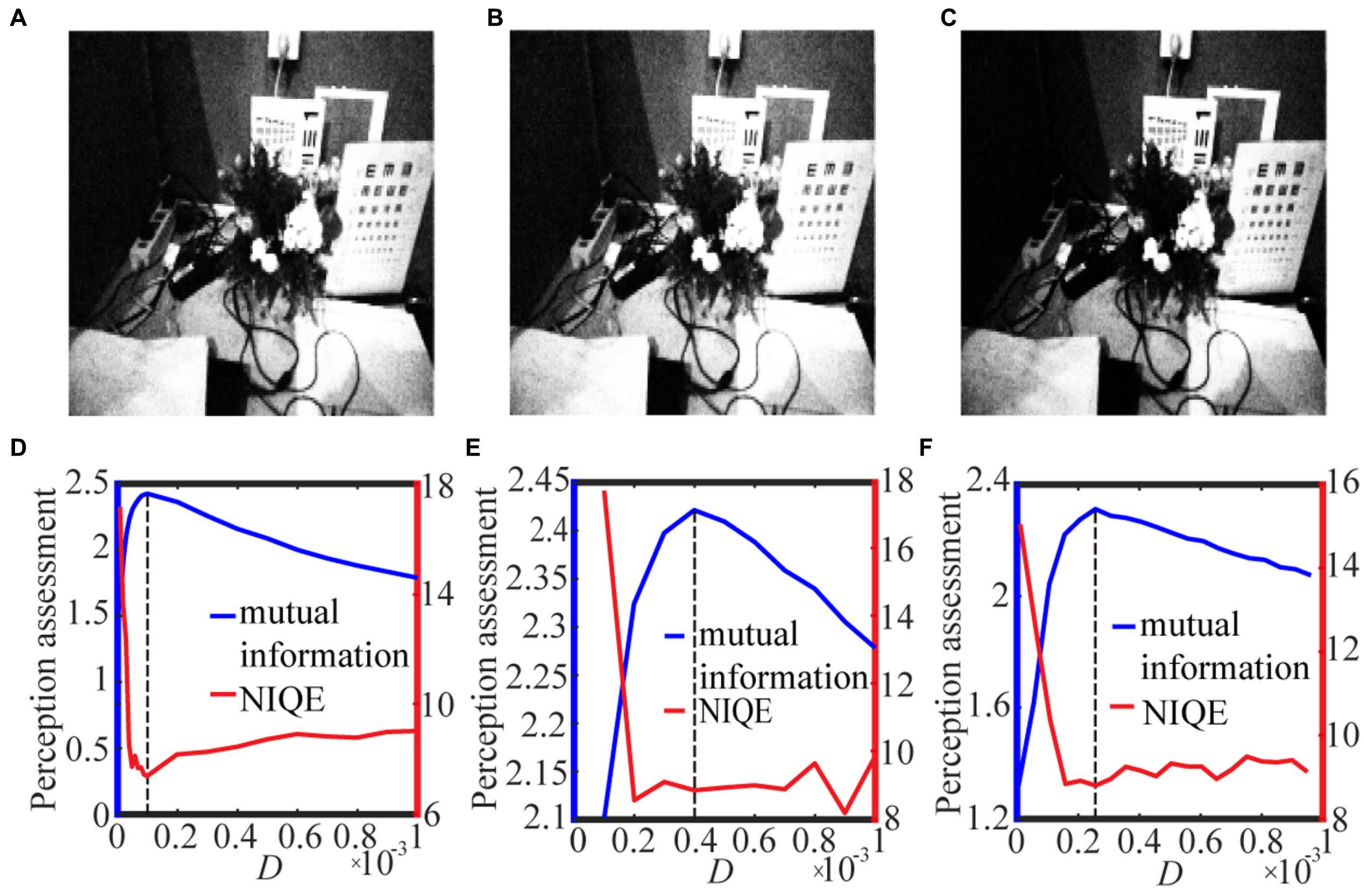
The best enhanced grayscale images selected by mutual information with spike thresholds **(A)** 0.5 quantile, **(B)** 𝛿 = 0.001 and **(C)** 𝛿 = 0.01 for Eq. (12). Their quantifying indexes which black dotted line denotes the optimal noise intensity corresponding to the maximal mutual information are plotted in panels **(D–F)**.

**Figure 9:**
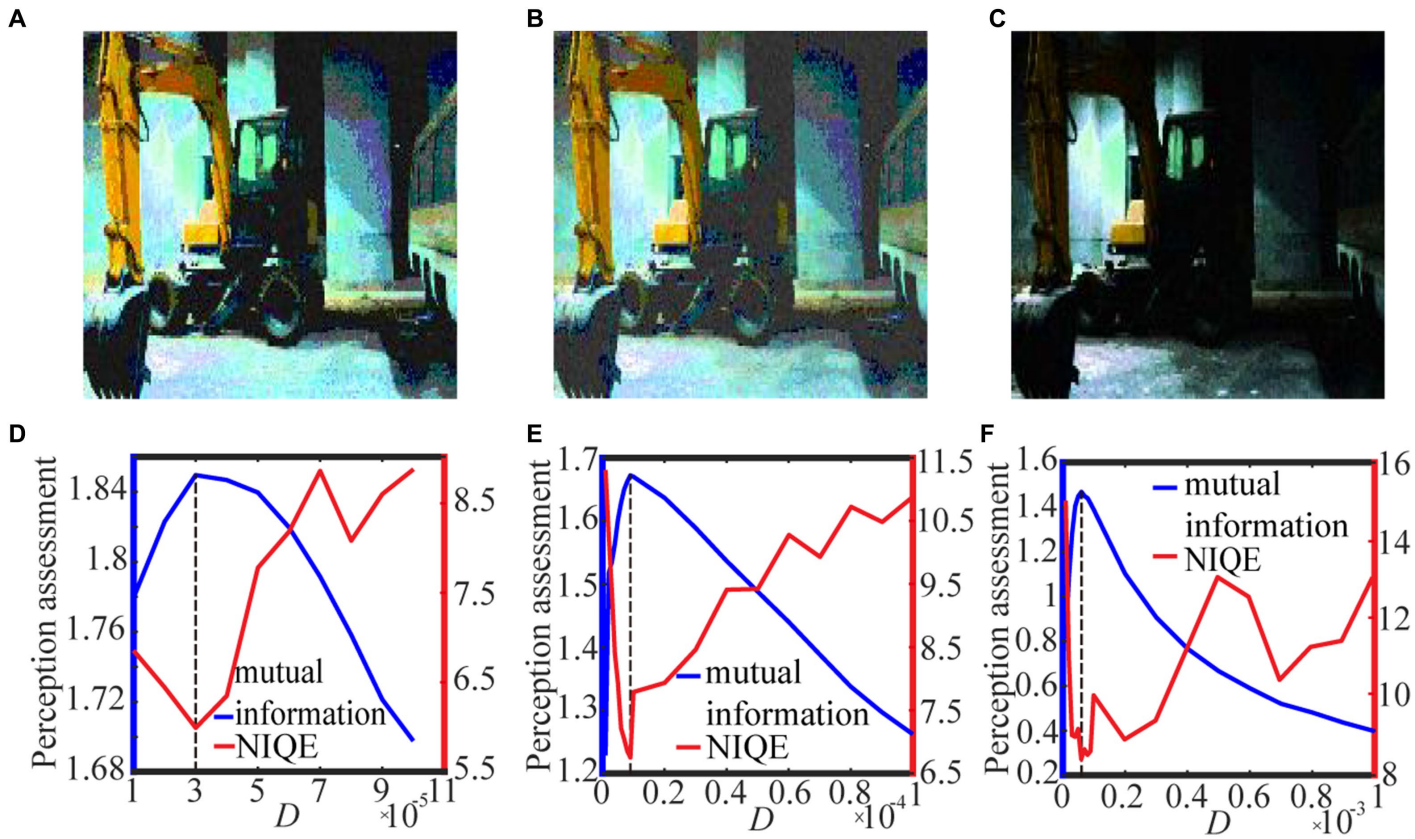
The best enhanced color images selected by mutual information with spike thresholds **(A)** 0.6 quantile, **(B)** 𝛿 = 0.001 and **(C)** 𝛿 = 0.01 for Eq. (13). Their quantifying indexes which black dotted line denotes the optimal noise intensity corresponding to the maximal mutual information are plotted in panels **(D–F)**.

As shown in Figure 2, the spike threshold has obvious influence on the aperiodic stochastic resonant effect. In fact, since the spike threshold always has important impact on neural activity and coding performance (Yu et al., 2005; Fu et al., 2020; Xu et al., 2022), it is also a critical parameter for the spiking neural network based visual perception. In the previous subsections, we choose some quantile of the relevant histogram of the original images as the spike threshold, and the results have showed that the metric of mutual information is more capable of picking out the best enhanced image. To strengthen this point, let us take a comparative perspective. To this end, let us attempt another threshold strategy (Reinhard et al., 2002), which takes a log-average luminance as threshold, namely


(12) $$V_{th}=\exp\left(\frac{\underset{m,n}{\sum }\log\left(\delta +Gray\right)}{m*n}\right)$$

for grayscale images and


(13) $$V_{th}=\exp\left(\frac{\underset{m,n}{\sum }\log\left(\delta +L\right)}{m*n}\right)$$

for color images. We remark that in Eqs. (12) and (13), a small positive number δ is introduced to avoid vanishing antilogarithm. In Figures 8, 9 we show the test results with the best quantile threshold and the log-average luminance threshold with different delta value.

For the grayscale image case, Figure 8 shows the variation of the target image and the assessment index under different spike thresholds. As seen from Figures 8A–C, the threshold change does not have much impact on the enhanced image, but it tends to be difficult for us to select the optimal noise intensity from the fluctuating curve of NIQE. By contrast, the curve of the image mutual information is smooth and sharp, as shown in Figures 8D–F, thus the mutual information metric is better for us to pick out the optimal noise intensity and then find the target image. Here, it is worthy to emphasize that the curve of the image mutual information is single-peaked, but the curve of NIQE has multiple local minimums. By checking the quality of the enhanced images at all the local optimal noise intensity, it is found that the enhanced image at the local optimal noise intensity, which is also the maximum point of the image mutual information, is the best enhanced image. This clearly demonstrates that the mutual information measure has advantage over the NIQE index in picking out the target image in the present visual perception research. That exactly manifests why we do this research. Additionally, it is observed that among the three strategies for the spike threshold, the mutual information curve corresponding to the one-half quantile has the most prominent peak, thus choosing a suitable quantile as the spike threshold (Xu et al., 2022) also has advantage over log-average luminance strategy (Reinhard et al., 2002).

For the color image case, Figure 9 shows the variation of the target image and the assessment index under different spike thresholds. It is clear that the optimal noise intensity with the image mutual formation is coincident with the optimal counterpart with the NIQE metric. This coincidence once again demonstrates both the metrics are effective for picking out the target image. Nevertheless, due to its smoothness, the image mutual information is more convenient than the NIQE metric, as suggested by the Figures. Additionally, it is evident that the target image (Figure 9C) is sensibly darker than the counterpart in Figures 9A,B, illustrating that the enhancement performance with the log-average threshold is sensitive to the choice of the small parameter. An inappropriate choice of the parameter delta in Eq. (13) can cause a bad enhancement. This of course is of inconvenience, and thus suggests that the quantile threshold strategy has merit from a viewpoint of antithesis.

## 4. Conclusion and discussion

We have revealed the phenomenon of aperiodic stochastic resonance in the conductance-based integrate-and-fire neuronal networks within the frame of information theory, and then we presented an improved spiking neural network based visual perception algorithm based on the principle of aperiodic stochastic resonance. In the improved algorithm, the image mutual information is adopted as a quantifying metric, since it can well measure the shared information between the input image stimulus and the enhanced target image. With the same trials in calculation, it was shown that the optimal noise intensity corresponding to the maximum of the mutual information coincides with one of the counterparts of the minimums of the NIQE index. More importantly, it was shown that the curve of the image mutual information *via* noise intensity is usually mono-peaked, sharper and smoother than that of the NIQE index *via* noise intensity. This illustrates that the applicability and advantage of the image mutual information over the frequently used index in visual perception. Additionally, with the numerical tests with the quantile of the image histogram as spike threshold scheme compared with those with the log-average luminance as spike threshold, it was further confirmed the mutual information index has more reliability than the NIQE index, since the results from the latter scheme are more sensitive to the increasing noise level. Nevertheless, note that the spike threshold is fixed during the entire implementation of the algorithm, so the quality of the best enhanced image might be further improved by an adaptive strategy, such as updating the threshold at each noise intensity by the quantile of the newly enhanced image. This is worthy to be explored in the near future. We also wish that the algorithm of this paper has application or inspiration in the relevant fields such as brain-machine interface, cosmic detection and target tracking in low-illumination environment.

## Data availability statement

The original contributions presented in the study are included in the article/supplementary material, further inquiries can be directed to the corresponding author.

## Author contributions

YK guided and sponsored the research. ZX did the simulation and algorithm design. YZ provided some data and attended the algorithm implementation. ZX and YK collaborated in writing and revising. All authors contributed to the article and approved the submitted version.

## Funding

This work was financially supported by the National Natural Science Foundation under grant nos. 11772241 and 12172268.

## Conflict of interest

The authors declare that the research was conducted in the absence of any commercial or financial relationships that could be construed as a potential conflict of interest.

## Publisher’s note

All claims expressed in this article are solely those of the authors and do not necessarily represent those of their affiliated organizations, or those of the publisher, the editors and the reviewers. Any product that may be evaluated in this article, or claim that may be made by its manufacturer, is not guaranteed or endorsed by the publisher.
